# A Challenging Conundrum; Learning Disability, Schizophrenia and Autism - a Case Report

**DOI:** 10.1192/j.eurpsy.2024.327

**Published:** 2024-08-27

**Authors:** F. Rouhani, G. Aperis, S. Digpal

**Affiliations:** General Internal Medicine, Queen Alexandra Hospital, Cosham, United Kingdom

## Abstract

**Introduction:**

Psychiatric disorders are common in patients with learning disabilities. There are also patients with the triad of autism, schizophrenia and learning disability. Patients with this background can be admitted to general hospitals for psychiatric or non-psychiatric reasons.

We are presenting a case who had a very complicated clinical course and her discharge planning was challenging.

**Objectives:**

The objective of this work was to show the challenges in the investigation, medical management, and discharge planning of the patients with concomitant learning disability, schizophrenia and autism.

**Methods:**

We scrutinized the patient’s casenotes, including blood results and all relevant imaging. We paid a particular attention to all the entries from the psychiatry team, general medical doctors, oncologists, learning disability team and discharge planners.

**Results:**

The lady had a protracted 4-month inpatient admission throughout which she was physically and verbally aggressive to hospital staff. She was deemed to lack capacity for hospital admission and treated in her best interests under Mental Capacity Act (MCA), frequently requiring sedation with Haloperidol and Lorazepam. Following consultation with the local Psychiatrist her medications were altered to: Risperidone 2 mg BD, Diazepam 5 mg OM, 5 mg afternoon and 10 mg evening, Procyclidine 5 mg BD, Chlorpromazine 25 mg BD, Promethazine 25 mg OD PRN, and Midazolam 10 mg buccal PRN.

A change in her clinical condition was noted by the Psychiatry team; increased agitation, confusion and dysarthria. A repeat blood test was advised, due to patient refusal this took weeks to achieve despite the use of buccal Midazolam following Anaesthesiologist advice. Although blood tests were not significantly deranged, she was treated for presumed urinary tract infection with a course of antibiotics. A urine sample was unobtainable.

She reported right breast pain and underwent a mammogram. This showed a hypoechoic lesion 8x7x9 mm. Following consultation with a Breast Surgeon and Oncologist, Letrozole was replaced with Tamoxifen. The Psychiatric team concluded that the clinical deterioration with dysarthria was related to anxiety, associated with Autistic patients. The movement disorder was deemed secondary to antipsychotics exacerbated by stress and anxiety.

The patient’s discharge planning was complex. She was declined by numerous care homes. At time of writing, she remains an inpatient.

**Conclusions:**

The management of patients with a triad of Learning Disability, Schizophrenia and Autism is extremely difficult, particularly within an acute medical setting. Physical deterioration could be related to medication adverse effects or change in environment. Anxiety and stress are linked to these conditions. The challenging behaviour that these patients often have will make discharge planning very difficult as specialised care homes to accommodate these unique patients are very limite

**Disclosure of Interest:**

None Declared

